# Animal Intelligence*Paper read at a meeting of Veterinary Society, Ontario Veterinary College, Toronto, February 3, 1891.

**Published:** 1891-03

**Authors:** H. Vulliamy

**Affiliations:** Veterinary Student, Ontario Veterinary College


### ANIMAL INTELLIGENCE.*

By H. Vulliamy.

Veterinary Student, Ontario Veterinary College.

In considering the intelligence of animals it would be well first to thoroughly understand the difference between instinct and reason.

To explain this in as few words as possible I would say that an action performed for the purpose of gaining the object it attains, is directed by reason. An action having for its object, the satisfying of some inward desire, and not for the purpose of accomplishing the object it ultimately attains, is the result of instinct. To make this still plainer, we will take for example an action with which we are all familiar, namely, eating. We, as a rule, eat, to satisfy a craving we have and not to support life ; therefore we eat instinctively.

The chief object of this essay, is to show the difference between the mental capacity of man and that of the domestic animals.

*Paper read at a meeting of Veterinary Society, Ontario Veterinary College, Toronto, February 3, 1891.

Dr. Sexton, who has given this subject a great deal of thought, claims for men four instinctive and three reasoning powers which animals do not possess. I use the term animal, as distinguished from human. The first instinctive power which man alone possesses is, "A knowledge that there is a Supreme Being." No one would dispute the fact that animals have no such instinct. Its existence in humanity however, might be questioned by some; but history shows us plainly that every nation or race of people, worshiped a Supreme Power in some form. Instinct told them that there was a something more powerful than they, and which was able to control them. Some connected it with the heavens, others with animals, and so on. To-day there are men in civilization who would wish to think otherwise; but their very perseverance in endeavoring to find argument to extinguish this instinct, proves its existence and the fallacy of their theories. The second instinctive power to be mentioned is this, "An instinctive fear of death." The opinion may be held by some that animals do fear death, yet if one hundred animals were placed in a row and killed, one by one, in such a way that no blood was shed and no pain was shown, the hundredth animal would be no more frightened than the first. Animals fear blood, and are angered or frightened when they see another in pain; but knowing nothing of the future, they are unable to fear that which is ahead, and cannot realize the process of death.

The third instinct to be spoken of is, "An instinctive fear of punishment when a wrong is committed." The proverbial "guilty conscience" we possess cannot be gainsayed, for we have all experienced it more or less. It might be claimed by some people that animals also possess this instinct. Fear of punishment may be connected with a certain action; but the animal does not feel that the action is wrong in itself, until punished for it.

Lastly, "Man has an instinctive desire to see justice done." However bad a man may be, if he hears of an injustice committed to another, instinctively he sides with the aggrieved. Among the brute creation we find the reverse taking place, for instance, the leading animal in a herd never excites indignation by his brutality.

We now arrive at the reasoning powers possessed only by humanity. In discussing the intelligence of animals, many persons, at the outset, would question the propriety of the term.

Man has so long arrogated the exclusive possession of a mind, or at least of a mind capable of rational reflection, that he is reluctant to concede the fact of its possession by the lower order of animal life. Those acts, which in the brute creation seem to proceed from the action of a power analogous to human intelligence, it has been usual to ascribe to this irrational faculty called instinct, a power, unvariable and despotic in its actions, but in no degree the result of reflection, some metaphysicians going so far as to assert, that the actions of animals are purely automatic, the difference, in this respect, between them and the automation moved by springs, being, that the power possesses a consciousness of their acts which the latter does not. Facts in myriads exist which challenge the correctness of such a theory and point to the existence, at least, in its embryonic condition, of a mind, capable of thought, and to a limited degree, of reflection and comparison, with the ability to deduce conclusions from the facts it considers. This intelligence varies greatly in different animal races, in some species being scarcely perceptible, while in others it is too conspicuous to be ignored. It is the absence of the power of speech in animals which leaves us in doubt as to the exact degree of intelligence possessed by them.

If when a farmer says to his dog: Carlo! the cows are in the grain. Turn them out! the dog should turn his head and say, "Yes sir, I will have them out in a moment," there would be no doubt of the intelligent interchange of thought. But the fact of his carrying out the order, which in the supposed case he would express, proves as conclusively his comprehension and his purpose to obey. The horse or dog, however fully he may understand the directions he receives, can give no other response than by his acts, and to words of praise or censure he can reply only by signs; these are already understood by us and show that our meaning is comprehended by the animal, thus proving a real interchange of thought. To test the existence and extent of intelligence, we must determine the capacity for comprehending thought. We recognize this capacity in a child long before it can express itself in language. Its dawn is seen as the infant learns to associate certain articulate sounds with certain persons, acts or things, to distinguish the meaning of tones which encourage, restrain, or chide it. It is only after twelve months or more of constant tuition, lovingly and intelligently given, that children begin to express in language the thoughts which are awakened by acts, yet the comprehension is as evident, the response as apparent, the whole mental process being in action long before.

The same test which proves the intelligence in a child demonstrates its existence in animals. There is a similar power of comprehending the wishes expressed, by associating certain articulate sounds with certain acts required, as well as an equal recognition of the tones of voice, by which, approval, reproof or anger are made known ; but lacking the organs of speech they are debarred and ever must be, from any, except the most limited interchange of thought. For this reason, attentive study is needed in ascertaining the extent to which they comprehend and respond to the intelligence which addresses them.

In domesticated animals, especially those which are trained for the use of man, the action of intelligence may be clearly traced ; it is demonstrated by the ease and certainty by which they can be educated. It is seen in the readiness with which many receive and act upon ideas communicated to them ; and in a multitude of instances, the mental process is evident, by which they have independently reached conclusions, rationally deduced from facts of their previous knowledge.

A circumstance is related of a terrier, who had been temporarily left by his master in the care of a Mr. Langford, of St Albans, England. The lady owned a large house dog, which, disliking the presence of the stranger quarreled with him, biting and severely wounding him, after which the terrier disappeared ; but in a few days he returned accompanied by a powerful mastiff, when both together fell upon the original assailant, whom they nearly killed. The mastiff was a watch-dog in his master's house, more than a day's journey distant, and had been brought by the terrier for the sole purpose of avenging the injury he had recieved, after which they left in company and proceeded together to their home.

Here was displayed a power of combining ideas and communicating them to one of his kind, when the two acted upon the plan they had preconcerted.

The elephant, though one of the clumsiest of animals, exhibits marks of high intelligence and evidently understands the language in which he is addressed. He can be stimulated to wonderful exertion by promise of a reward.

I have seen, says a French writer, two occupied in beating down a wall which their keeper had desired them to do and encouraged them by a promise of fruits and brandy.

They were left alone and continued at their work until it was accomplished. "When a reward is promised to an elephant," says the same writer, "it is dangerous to disappoint him, as he never fails to avenge the insult." Nothing of this could occur without an understanding of the language.

It may be said that the tones of the voice, rather than the words, are what the animal understands, yet a dog knows his name however spoken and a horse understands a whole vocabulary of orders. But the intelligence which comprehends the meaning of a tone, is not less, than that required to understand a word or sentence.

Having discussed the existence of intelligence in the brute creation, it is now necessary to compare the mental capacity of man and beast. The three faculties which man possesses and which animals are totally devoid of, are: First, "the power of planning for the future." With the animal the affairs of the moment alone engrosses the mind. Secondly, "Man is able to imagine, animals cannot."

To build castles in the air is an ability man alone possesses. Though dreams of the past may disturb the slumbers of the brute creation, they can never have dreams of the future dashed to the ground. They live entirely in the present! Thirdly, "Animals cannot deal with anything in the abstract." An abstract idea in methaphysics, is an idea separated from a complex object, or from other ideas which naturally accompany it, for instance, an animal could not consider the solidity of a stone, apart from the color and shape.

Of these mental faculties animals are totally devoid. Man possesses the other reasoning powers more fully developed; but in animals they undoubtedly exist, in a greater or less degree, good training in some cases showing them to great advantage.

The systems of Pratt, Rockwell and others, have done a great deal to raise the standard of the equine race, teaching, as they do, that horses require to be educated and not tamed. The good results of these methods are seen every day.

